# Posterior dislocation of the Oxford knee meniscal bearing: a treatment option

**DOI:** 10.1007/s10195-013-0250-2

**Published:** 2013-06-25

**Authors:** S. Tibrewal, H. Pandit, P. McLardy-Smith, S. B. Tibrewal, D. W. Murray

**Affiliations:** 1 St Bartholomew’s and the Royal London Hospitals, London, UK; 2 Orthopaedic Department, Queen Elizabeth Hospital, Woolwich, UK; 3 Nuffield Orthopaedic Centre, Oxford, UK; 4 59 Elmstead Lane, Chislehurst, Kent BR7 5EQ UK

**Keywords:** Unicondylar knee replacement, Bearing dislocation, Oxford knee

## Abstract

Unicompartmental knee replacement (UKR) is now established as a treatment for medial compartment arthritis. The Oxford UKR (Biomet Orthopedics, Inc, Warsaw, IN, USA) has a mobile-bearing system, which minimizes wear. This has been shown to provide excellent long-term results. Dislocation of the mobile-bearing device is rare with an incidence of 1 in 200 (0.5 %). The treatment usually involves exploration of the knee through the original anteromedial incision, removal of the dislocated bearing and rectification of the underlying cause for the dislocation. We describe two cases of a posterior dislocation in which the mobile bearing could not be retrieved and was left in situ. In both cases a good outcome was achieved. We conclude that in extremely rare cases where a dislocated bearing has migrated posteromedially and cannot be retrieved, it can be left in place rather than exploring the joint acutely through a separate posterior incision.

## Introduction

Unicompartmental knee replacement (UKR) is an established treatment for end-stage medial compartment arthritis providing good pain relief and restoring function. The Oxford knee is the most widely-used UKR with long-term survival being comparable to that achieved with total knee replacement, provided that the indications and surgical technique are appropriate [1, 2]. The Oxford knee has a mobile bearing, which minimises wear but renders it vulnerable to dislocation. However, this is rare with an incidence of 0.5 % [1, 2]. The causes for dislocation include trauma, ligamentous injury, bearing impingement against retained posterior osteophytes, cement or anterior bone, mal-position of the components and loosening of components. The aim of this paper is to present our experience of an alternative option for an extremely rare situation that a surgeon may unexpectedly face.

## Case reports

We describe the management of two cases of posterior dislocation in which the mobile bearings could not be retrieved through a mini anteromedial approach. Rather than making a separate posterior approach, these displaced bearings were left in situ and a new one inserted providing a stable articulation. Below, we review the literature and discuss treatment strategies for this rare complication.

Both patients gave their consent prior to their inclusion in this report.

### Case 1

A 64-year old man underwent Oxford medial UKR in September 2004. He had previously had an arthroscopy of the same knee for a complex tear of the medial meniscus. The patient’s medical history included coronary artery bypass graft, atrial fibrillation, and right mid-foot fusion. He had been on a number of medications including Warfarin.

At the index procedure a medium-sized femoral component, 50 × 32 mm tibial tray, and a size 7 meniscal bearing provided a stable articulation. The knee remained relatively pain-free for 5 years until he sustained a twisting injury to his knee. Following this he developed severe pain and swelling in the knee and was unable to weight bear. He attended the A&E Department on the same day and was reviewed in the fracture clinic the following day. Radiographs demonstrated a posterior dislocation of the mobile bearing.

The patient consented to an exchange of bearing or revision total knee replacement. The knee was explored through the previous mini anteromedial incision. Both femoral and tibial components were found to be well-fixed with no macroscopic evidence of burnishing, abrasions or scratching over the metal surfaces. The anterior cruciate and collateral ligaments were intact. Intraoperatively, despite an extensive search, the dislocated bearing could not be retrieved. A trial reduction with a size 9 medium meniscus provided a stable articulation with no impingement, no varus or valgus instability, and no lift off of the trial meniscus. It was decided not to explore the knee through a posterior approach to retrieve the meniscus in order to avoid the added morbidity of a more invasive procedure. A definitive bearing was inserted and the wound was closed in layers. Total surgical time for the procedure was 1 h and no additional antibiotics were administered. The postoperative recovery was uneventful and the patient was mobilised, fully weight-bearing, without any difficulty. The patient's status was reviewed regularly in the outpatient clinic. Clinically he remained relatively pain-free and continued to enjoy a relatively normal function of the left knee for 2 years following insertion of the replacement bearing.

**Fig. 1:**
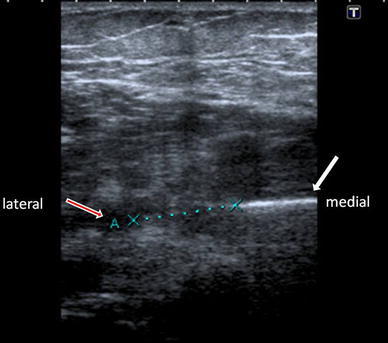
Ultrasound scan to monitor displaced meniscus with *medial arrow* showing position of dislodged meniscus and *lateral arrow* (*A*) indicating popliteal blood vessels

In order to ensure that the dislocated bearing was not migrating and endangering the neurovascular bundle in the popliteal fossa, we performed serial ultrasound scans. These scans demonstrated that the meniscus was located posteromedially approximately 2 cm below the joint line and some distance from the neurovascular bundle (Fig. 1). Its position remained unchanged. The patient did not wish to have any further surgery to retrieve the dislocated meniscal component, which would have required a posterior approach.

### Case 2

A 61-year old female patient treated at another centre independently by another surgeon underwent Oxford medial unicompartmental right knee replacement in September 2004. A medium-sized femoral component, size E tibial tray and a medium size 4 meniscal bearing provided a stable articulation. The patient was fit and healthy except for the history of Ménière’s disease.

Postoperatively the knee remained pain-free for 6 months following the original operation until she sustained a twisting injury to her knee. Following this she developed pain and swelling to the right knee and was unable to bear weight. Radiographs demonstrated a posterior dislocation of the bearing.

The patient consented to exploration of the knee, bearing retrieval/exchange or revision to total knee replacement. The knee joint was explored through the old mini anteromedial incision. Both femoral and tibial components were found to be well-fixed with no macroscopic evidence of burnishing, abrasions or scratching over the metal surfaces. The anterior cruciate ligament and collateral ligaments were intact. Intraoperatively, the dislocated bearing could not be retrieved through the mini anteromedial approach. A trial reduction with a size 7 medium meniscus provided a stable articulation with no impingement, no varus or valgus instability, and no lift off of the trial meniscus. It was decided not to explore the knee through a posterior approach to retrieve the meniscus. A definitive bearing was inserted and the wound was closed in layers. The knee joint was supported with wool and crepe bandages. Total surgical time for the procedure was 1 h and no additional antibiotics were administered.

The postoperative recovery was uneventful and the patient was mobilised, fully weight-bearing, without any difficulty. Her condition was reviewed in the clinic regularly. Clinically she remained relatively pain-free and continued to enjoy normal function of the right knee 4 years following surgery.

## Discussion

The invention of a mobile-bearing knee replacement introduced the complication of a dislocation of a mobile bearing [3]. However, following the introduction of instrumentation to accurately balance and tension the ligaments in the phase 2 and phase 3 design of the Oxford mobile-bearing knee, the incidence of this complication in the medial compartment is approximately 0.5 % [2]. The incidence in the lateral side is higher or the ligaments are more extensible. As a result, the use of a mobile bearing in the lateral compartment with a flat tibial component is not recommended [4, 5].

For a primary medial dislocation to occur there has to be both distraction of the joint surfaces and displacement of the bearing. Displacement is usually the result of impingement of the bearing against retained osteophytes [6], protruding cement or bone anterior to the femoral component. Distraction is likely to occur if there is damage to the medial collateral ligament (MCL) or if the flexion/extension gaps are not equal. The front of the meniscal bearing is about 5 mm higher than the deepest part of the bearing. So, a posterior dislocation is unlikely unless the bearing has spun resulting in a decrease in the entrapment.

The risk of the bearing spinning is now low with asymmetrical anatomic bearings. Primary dislocations usually occur early. Secondary dislocations tend to occur later and may be associated with loosening of the metal components. Traumatic dislocation, as described in our cases, has also been reported occasionally.

In our first case, the dislocation occurred 5 years after its original insertion following a twisting injury to the knee. At operation the components were found to be well-fixed. Radiographs confirmed the presence of a narrow radiolucent line around the tibial component, which we refer to as a “physiological radiolucency” [7], and significant degenerative changes in other compartments but the patient remains relatively pain-free.

In the second case, the dislocation occurred 6 months post index procedure, again following a twisting injury. Both components were secure. The second patient also remained relatively pain-free at the time of last follow-up.

In both cases, no cause for the dislocation was found except stretching of the MCL due to the traumatic twisting injury sustained by the patient.

Dislocation can occasionally be treated by manipulation and relocation of the meniscus. However, arthrotomy through the old anteromedial incision is almost always required to remove the bearing and to determine and rectify the cause of its displacement. The bearing can usually be retrieved through the anterior incision even if it has displaced to the back of the joint. However, in the cases above, the bearings could not be found through the anteromedial approach, as they had migrated well below the joint line. Although, a posterior approach can be used to retrieve the menisci, these two cases demonstrate that this is probably not essential. If the retained bearing did cause problems in the future, it could be removed with further surgery at that point.

For the definitive treatment of a dislocated bearing, the underlying cause of the primary or secondary dislocation needs to be addressed. Damage to the extraarticular surface of the bearing suggests impingement. Impingement should be rectified by removing excess bone or cement. Slight imbalance of the knee can be accepted. If the bearing has been dislocated for some time, there may be damage to the metal surfaces of the components. This damage tends to be local flattening of the femoral surface or a local concavity in the tibial surface. Since the damaged area is smooth it can be ignored. Following a trial reduction to ensure the bearing tracks satisfactorily, a new anatomic bearing of correct size should be inserted. Care should be taken to avoid over tightening the knee as this will not prevent further dislocation and may in fact cause other problems.

If there is gross imbalance of the knee joint, it is possible to remove the tibial plateau and insert a fixed-bearing component. Similarly, if a femoral component is loose and there is minimal bone damage, a new femoral component can be inserted. However, in general it is felt that if the cause of dislocation cannot be corrected and further dislocation is inevitable, it would be better to convert the UKR to a total-knee replacement.

In general, treatment of a primary dislocation by addressing the underlying problem and replacing the bearing of the same size or slightly thicker is successful, and recurrent dislocation does not usually occur.

This case report demonstrates that in the event of a posterior dislocation of the bearing, which cannot be retrieved through the original mini-arthrotomy, it is probably safe to leave it in situ and simply replace the bearing.
